# Factors associated with temporary pacing insertion in patients with inferior ST-segment elevation myocardial infarction

**DOI:** 10.1371/journal.pone.0251124

**Published:** 2021-05-03

**Authors:** Tomonobu Yanase, Kenichi Sakakura, Hiroyuki Jinnouchi, Yousuke Taniguchi, Kei Yamamoto, Takunori Tsukui, Masaru Seguchi, Hiroshi Wada, Hideo Fujita

**Affiliations:** Division of Cardiovascular Medicine, Saitama Medical Center, Jichi Medical University, Shimotsuke, Japan; Erasmus Medical Centre: Erasmus MC, NETHERLANDS

## Abstract

**Background:**

High-degree atrioventricular block (HAVB) is a prognostic factor for survival in patients with inferior ST-segment elevation myocardial infarction (STEMI). However, there is little information about factors associated with temporary pacing (TP). The aim of this study was to find factors associated with TP in patients with inferior STEMI.

**Methods:**

We included 232 inferior STEMI patients, and divided those into the TP group (n = 46) and the non-TP group (n = 186). Factors associated with TP were retrospectively investigated using multivariate logistic regression model.

**Results:**

The incidence of right ventricular (RV) infarction was significantly higher in the TP group (19.6%) than in the non-TP group (7.5%) (p = 0.024), but the incidence of in-hospital death was similar between the 2 groups (4.3% vs. 4.8%, p = 1.000). Long-term major adverse cardiovascular events (MACE), which were defined as a composite of all-cause death, non-fatal myocardial infarction (MI), target vessel revascularization (TVR) and readmission for heart failure, were not different between the 2 groups (p = 0.100). In the multivariate logistic regression analysis, statin at admission [odds ratio (OR) 0.230, 95% confidence interval (CI) 0.062–0.860, p = 0.029], HAVB at admission (OR 9.950, 95% CI 4.099–24.152, p<0.001), and TIMI-thrombus grade ≥3 (OR 10.762, 95% CI 1.385–83.635, p = 0.023) were significantly associated with TP.

**Conclusion:**

Statin at admission, HAVB at admission, and TIMI-thrombus grade ≥3 were associated with TP in patients with inferior STEMI. Although the patients with TP had the higher incidence of RV infarction, the incidence of in-hospital death and long-term MACE was not different between patients with TP and those without.

## Introduction

Ischemic heart disease (IHD) remains the number 1 cause of death globally, although the complications and mortality rates of acute myocardial infarction (AMI) have declined over the past 20 years owing to the progress of coronary intervention and optimal medical therapy [1, 2]. In order to improve clinical outcomes in patients with AMI, it is essential to collect more specific data about each complication of AMI. High-degree atrioventricular block (HAVB) is a common complication following ST-segment elevation myocardial infarction (STEMI), the incidence of which is reported to be 2.7 to 19.6% [3–7]. Each complication of AMI depends on the size and anatomic location of the infarction, and HAVB is more common in inferior STEMI [3, 4].

Temporary pacing (TP), which is indicated for symptomatic or hemodynamically significant bradycardia, is indispensable for some patients with bradycardia due to inferior STEMI [8]. Insertion of a transvenous TP is a time-consuming procedure, and has its own complications including bleeding, thrombosis, infection, delirium, arrhythmia and cardiac perforation [9–11]. When a patient with inferior STEMI comes to a catheter laboratory, an interventional cardiologist has to decide whether to insert a TP within a short time period. Several groups reported factors associated with HAVB in patients with inferior STEMI, but there is little information regarding factors associated with TP in patients with inferior STEMI [12]. HAVB does not necessarily require TP, whereas bradycardia without HAVB may require TP in certain situations. In the present study, we aimed (1) to find factors associated with TP in patients with inferior STEMI, and (2) to compare clinical outcomes between those who received TP and those who did not.

## Methods

### Study design

This was a retrospective, single center study. We reviewed consecutive AMI patients from hospital records in our medical center from January 2015 to December 2019. The inclusion criteria were (1) AMI due to right coronary artery (RCA) and (2) STEMI. The exclusion criteria were (1) in-hospital onset, (2) no revascularization, (3) underwent coronary artery bypass graft surgery to the culprit lesion of AMI, (4) second or more than second AMI during the same study period, (5) had undergone permanent pacing implantation before the AMI, and (6) TP was not activated, because pacing was inserted for prophylactic purpose. Final study population was divided into a TP group and a non-TP group according to the insertion of TP. Clinical characteristics were compared between the TP and non-TP groups. Our primary interest was to find factors associated with TP using multivariate logistic regression model. We also examined major adverse cardiovascular events (MACE) until September 30^th^ 2020. MACE were a composite of all-cause death, non-fatal myocardial infarction (MI), target vessel revascularization (TVR) and readmission for heart failure. We defined the admission day as the index day in this follow-up analysis. This study was approved by the institutional review board of Saitama Medical Center, Jichi Medical University (S20-132), and written informed consent was waived because of the retrospective study design.

### Definitions

AMI was defined according to the universal definition [3, 13]. Diagnostic ST elevation was defined as new ST elevation at the J point in at least two contiguous leads of 2 mm (0.2 mV), and the AMI patients with ST elevation were diagnosed as STEMI [14]. HAVB was defined as the presence of either Mobitz II second-degree AV block or third-degree AV block [15]. Sick sinus syndrome (SSS) was defined as sinus bradycardia, sinoatrial pause of 3 seconds or more, sinoatrial exit block, or sinus arrest [16]. Bradycardia was defined as a rate below 60 beats per minute [17]. Hypertension was defined as systolic blood pressure (SBP) >140 mmHg, diastolic blood pressure >90 mmHg, or medical treatment for hypertension [18, 19]. Diabetes mellitus was defined as hemoglobin A1c (HbA1c) ≥6.5% or treatment for diabetes mellitus [19, 20]. Dyslipidemia was defined as total cholesterol ≥220 mg/dL, low-density lipoprotein cholesterol ≥140 mg/dL, or treatment for dyslipidemia [19, 20]. We also calculated estimated glomerular filtration rate (eGFR) using serum creatinine (Cr), age, weight, and gender according to the following formula: eGFR = 194×Cr^−1.094^×age^−0.287^ (male), or eGFR = 194×Cr^−1.094^×age^−0.287^×0.739 (female) [21]. Shock was defined as SBP <90 mmHg, vasopressors required to maintain blood pressure, or attempted cardiopulmonary resuscitation [19, 20]. Left ventricular ejection fraction (LVEF) was measured using a modified Simpson method. Echocardiography was evaluated during the index hospitalization. Right ventricular (RV) infarction was defined as ST-segment elevation in V4R (1mm) or abnormal RV wall motion on echocardiography, accompanying clinical symptoms such as hypotension [20].

Quantitative coronary angiography parameters were measured using a cardiovascular angiography analysis system (QAngio XA 7.3, MEDIS Imaging Systems, Leiden, Netherlands). The lesion length and reference diameter were measured. Intracoronary thrombus was angiographically identified by the thrombolysis in myocardial infarction (TIMI) thrombus grade, and was scored in 5 grades as previous studies reported [22]. G0, no thrombus present; G1, possible thrombus present, with angiographic characteristics suggestive of thrombus but not diagnostic of thrombus (i.e., reduced contrast density, haziness, irregular lesion contour or a smooth convex meniscus at the site of total occlusion); G2, definite thrombus present, with greatest dimensions ≤0.5 the vessel diameter; G3, definite thrombus present, with greatest linear dimension >0.5 but <2 vessel diameters; G4, definite thrombus present, with the largest dimension ≥2 vessel diameters; G5, total occlusion, the size of thrombus cannot be assessed [22]. Dominant RCA was defined when RCA supplied circulation to both the inferior portion of the interventricular septum via the right posterior descending artery and the atrioventricular node via the right postero-lateral branch [23, 24]. Balanced RCA was defined when RCA supplied circulation to only the right posterior descending artery [23]. In addition, we counted the number of atrioventricular node branch (#4AV) to evaluate the size of RCA. We defined a #4AV artery as an artery branched from atrioventricular groove with ≥1 mm diameter.

### Statistical analysis

Data are presented as a percentage for categorical variables and the mean ± SD for continuous variables. Categorical variables were compared using Pearson’s χ2 test or Fisher’s exact test. The Shapiro-Wilk test was conducted to determine whether the continuous variables were normally distributed. Normally distributed continuous variables were compared between the groups using the unpaired Student’s t-test. Otherwise, continuous variables were compared using the Mann-Whitney U test. Kaplan-Meier survival analysis was performed with respect to MACE, and the difference between the two survival curves was compared by the log rank test. Furthermore, we performed multivariate logistic regression analysis to investigate factors associated with TP. Univariate logistic regression analysis was performed to identify variables that had marginal association with TP, and all variables that had marginal association (P < 0.20) in univariate analysis were adopted as independent variables in multivariate logistic regression analysis. Moreover, when there are ≥2 similar variables, only one variable was entered into the multivariable logistic model to avoid multi-collinearity. Odds ratio (OR) and 95% confidence interval (CI) were calculated. All reported P-values were determined by two-sided analysis, and P-values <0.05 were considered significant. All analyses were performed with IBM SPSS statistics version 25 (Chicago, IL, USA).

## Results

**Fig 1:**
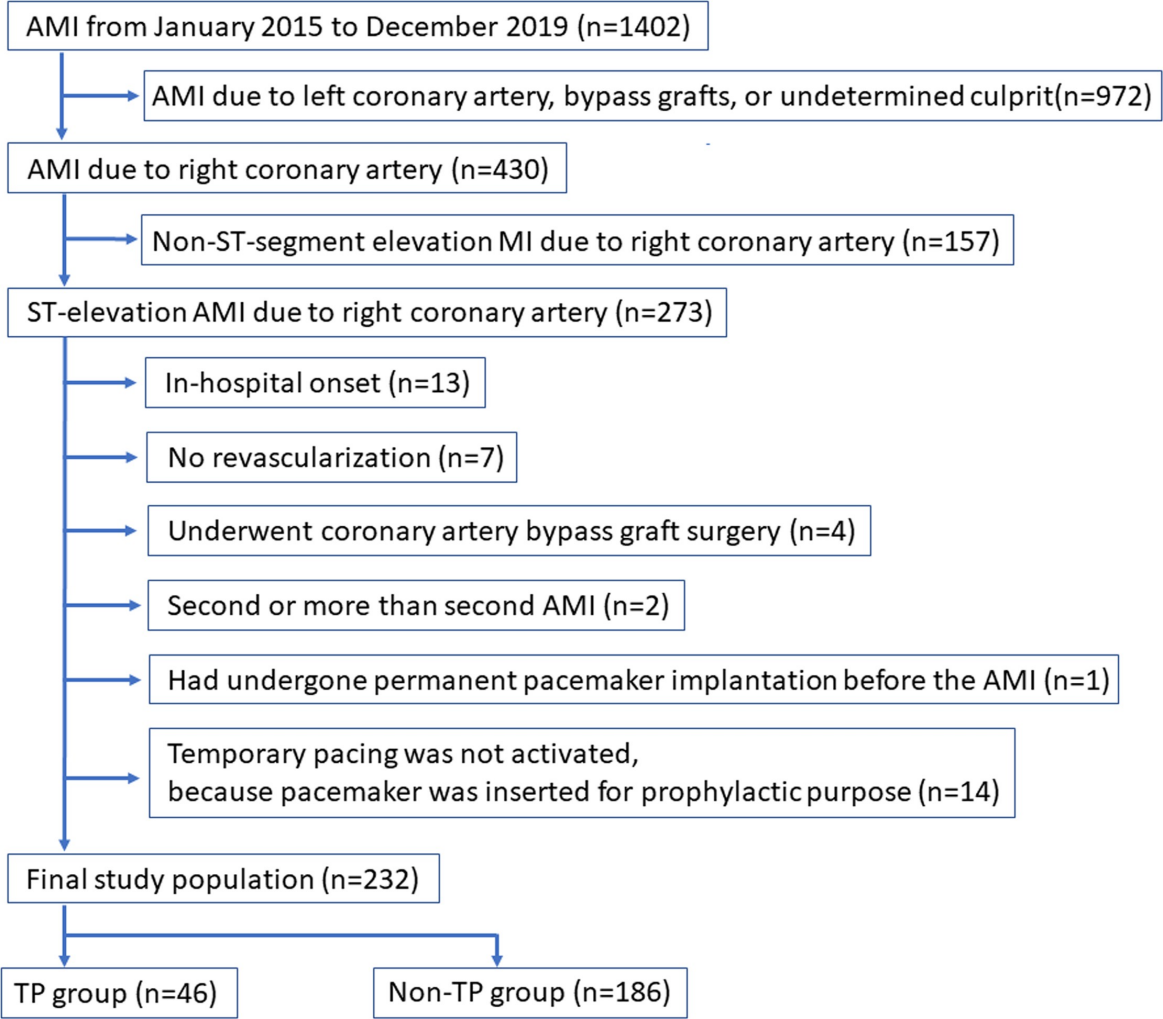
Study flow chart. AMI indicates acute myocardial infarction.

Among 1402 patients admitted to our medical center from January 2015 to December 2019, a total of 232 patients were included as the final study population, and were divided into the TP group (n = 46) and the non-TP group (n = 188) (Fig 1).

The details of the TP group are shown in Table 1. Of 46 patients with TP, 43 patients (93.5%) received TP before the revascularization, and 38 patients (82.6%) removed TP just after the revascularization. The reasons for TP were HAVB (69.6%), SSS (23.9%), atrial fibrillation with bradycardia (4.3%), and bradycardia (details unknown) (2.2%). The more detail regarding the catecholamine and mechanical circulatory support use by each bradyarrhythmias is shown in S1 Table.

**Table 1:** Details of the temporary pacing group.

	TP group (n = 46)
Placement of temporary pacing	
Before CAG, n (%)	21 (45.7)
After CAG and before PCI, n (%)	22 (47.8)
During PCI, n (%)	3 (6.5)
Removal of temporary pacing	
After PCI, n (%)	38 (82.6)
The next day, n (%)	2 (4.3)
3^rd^ hospital day, n (%)	2 (4.3)
4^th^ hospital day, n (%)	1 (2.2)
7^th^ hospital day, n (%)	1 (2.2)
9^th^ hospital day, n (%)	1 (2.2)
13^th^ hospital day, n (%)	1 (2.2)
Reason for temporary pacing	
High-degree atrioventricular block, n (%)	32 (69.6)
Sick sinus syndrome, n (%)	11 (23.9)
Atrial fibrillation with bradycardia, n (%)	2 (4.3)
Bradycardia (details unknown)	1 (2.2)

Abbreviations: *CAG* coronary angiography, *PCI* percutaneous coronary intervention.

Table 2 shows the comparison of patients’ characteristics between the 2 groups. The patients who had statin at admission were significantly less in the TP group (6.5%) than in the non-TP group (26.0%) (p = 0.004). The incidence of shock at admission was greater in the TP group (32.6%) than in the non-TP group (12.9%) (p = 0.001). The frequency of HAVB at admission was significantly higher in the TP group (50.0%) than in the non-TP group (5.9%) (p<0.001). In the non-TP group, eleven patients presented with HAVB were not treated with TP, because junctional escape rhythm was observed in 2 patients, and HAVB was transient in 9 patients.

**Table 2:** The comparison of patient’s clinical characteristic between the temporary pacing group and the non-temporary pacing group.

	**All (n = 232)**	**TP group (n = 46)**	**Non-TP group (n = 186)**	***P* value**
Age, year	69.8 ± 13.9	69.4 ± 15.1	69.9 ± 13.6	0.948
Male, n (%)	176 (75.9)	36 (78.3)	140 (75.3)	0.671
Height, cm	161.8 ± 9.9 (226/232)	161.8 ± 9.3	161.8 ± 10.1 (180/1867)	0.935
Weight, kg	63.1 ± 14.0 (230/232)	63.1 ± 17.2	63.1 ± 13.2 (184/186)	0.996
Current smoker, n (%)	91/227 (40.1)	19 (41.3)	72/181 (39.8)	0.850
Hypertension, n (%)	164/230 (71.3)	32 (69.6)	132/184 (71.7)	0.771
Diabetes mellitus, n (%)	93/230 (40.4)	16 (34.8)	77/184 (41.8)	0.382
HbA1c, %	6.7 ± 1.6 (221/232)	6.7 ± 1.5 (45/47)	6.7 ± 1.6 (176/186)	0.883
Dyslipidemia, n (%)	119/227 (52.4)	17/45 (37.8)	102/182 (56.0)	0.028
Serum creatinine, mg/dl	1.3 ± 1.8	1.5 ± 1.6	1.3 ± 1.8	0.016
eGFR, mL/min/1.73m^2^	64.1 ± 35.7	54.9 ± 25.7	66.5 ± 37.4	0.037
eGFR <60, n (%)	111 (47.8)	26 (56.5)	85 (45.7)	0.188
Chronic renal failure on hemodialysis, n (%)	12 (5.2)	2 (4.3)	10 (5.4)	1.000
Hemoglobin, g/dL	13.2 ± 2.0	12.8 ±2.1	13.3 ± 2.0	0.198
C-reactive protein, mg/L	1.7 ± 4.0 (229/232)	1.8 ± 3.6 (45/46)	1.7 ± 4.0 (184/186)	0.460
Brain natriuretic peptide, pg/ml	254.2 ± 454.4 (219/232)	307.4 ± 490.0 (45/46)	240.4 ± 445.2 (174/186)	0.885
History of previous myocardial infarction, n (%)	25 (10.8)	2 (4.3)	23 (12.4)	0.181
History of previous CABG, n (%)	1 (0.4)	0 (0.0)	1 (0.5)	1.000
History of previous PCI, n (%)	31 (13.4)	3 (6.5)	28 (15.1)	0.128
Killip classification				0.508
1 or 2, n (%)	193 (83.2)	36 (78.3)	157 (84.4)	
3, n (%)	6 (2.6)	1 (2.2)	5 (2.7)	
4, n (%)	33 (14.2)	9 (19.6)	24 (12.9)	
Cardiac arrest at out of hospital, n (%)	10 (4.3)	1 (2.2)	9 (4.8)	0.691
Shock at admission, n (%)	39 (16.8)	15 (32.6)	24 (12.9)	0.001
Pre-hospital syncope, n (%)	28 (12.1)	11 (23.9)	17 (9.1)	0.006
High-degree atrioventricular block at admission, n (%)	34 (14.7)	23 (50.0)	11 (5.9)	< 0.001
Atrial fibrillation, n (%)	9 (3.9)	3 (6.5)	6 (3.2)	0.386
Systolic blood pressure at admission, mmHg	127.2 ± 31.1 (228/232)	114.0 ± 27.9 (45/46)	130.5 ± 31.1 (183/186)	0.002
Diastolic blood pressure at admission, mmHg	74.4 ± 20.4 (227/232)	65.0 ± 21.6 (45/46)	76.7 ± 19.5 (182/186)	< 0.001
Heart rate at admission, bpm	69.9 ± 21.6 (231/232)	51.9 ± 15.6	74.3 ± 20.6 (185/186)	< 0.001
Admission route				0.024
Referred from another hospital, n (%)	89 (38.4)	12 (26.1)	77 (41.4)	
Walk in, n (%)	10 (4.3)	0 (0.0)	10 (5.4)	
Ambulance transport, n (%)	133 (57.3)	34 (73.9)	99 (53.2)	
From the onset				0.886
Within 24 hours, n (%)	201 (86.6)	39 (84.8)	162 (87.1)	
Over 24 hours, n (%)	19 (8.2)	4 (8.7)	15 (8.1)	
Undetermined, n (%)	12 (5.2)	3 (6.7)	9 (4.8)	
Medical therapy at admission				
Aspirin, n (%)	33/229 (14.4)	3 (6.5)	30/183 (16.4)	0.088
Thienopyridine, n (%)	18/228 (7.9)	1 (2.2)	17/182 (9.3)	0.133
Chronic statin therapy, n (%)	50/227 (22.0)	3 (6.5)	47/181 (26.0)	0.004
Calcium channel blocker, n (%)	79/227 (34.8)	18 (39.1)	61/181 (33.7)	0.490
ACE inhibitors or ARBs, n (%)	75/227 (33.0)	15 (32.6)	60/181 (33.1)	0.945
Beta-blockers, n (%)	21/227 (9.3)	3 (6.5)	18/181 (9.9)	0.581
Diuretics, n (%)	19/228 (8.3)	4 (8.7)	15/182 (8.2)	1.000
Oral antidiabetic, n (%)	47/229 (20.5)	9 (19.6)	38/183 (20.8)	0.857
Insulin, n (%)	15/230 (6.5)	5 (10.9)	10/184 (5.4)	0.188
Warfarin, n (%)	4/226 (1.8)	1 (2.2)	3/180 (1.7)	1.000
DOAC, n (%)	2/226 (0.9)	1 (2.2)	1/180 (0.6)	0.366

Data were expressed as mean ± SD or numbers (percentages). A Student’s *t* test was used for normally distributed continuous variables, and Mann–Whitney *U* test was used for abnormally distributed continuous variables. A Chi-square test was used for categorical variables.

Abbreviations: *ACE inhibitors* angiotensin-converting enzyme inhibitor, *ARB* angiotensin receptor blockers, *CABG* coronary artery bypass grafting, *CK* creatine kinase, *CK-MB* creatine kinase MB, *DAPT* dual antiplatelet therapy, *eGFR* estimated glomerular filtration rate, *HDL* high-density lipoprotein, *LDL* low-density lipoprotein, *PCI* percutaneous coronary intervention, *DOAC* direct oral anticoagulants.

Table 3 shows the comparison of angiographic lesion and procedural characteristics between the TP and non-TP groups. There were significant differences between the 2 groups in the initial TIMI flow grade (p = 0.024) and TIMI thrombus grade (p = 0.001). The prevalence of patients who underwent thrombectomy was higher in the TP group (58.7%) than in the non-TP group (26.3%) (p<0.001). There were no significant differences in RCA dominance (p = 0.351), and the number of #4AV (p = 0.167) between the 2 groups.

**Table 3:** The comparison of lesion and procedural characteristic between the temporary pacing group and the non-temporary pacing group.

	**All (n = 232)**	**TP group (n = 46)**	**Non-TP group (n = 186)**	***P* value**
Number of narrowed coronary arteries				0.875
1, n (%)	88 (37.9)	18 (39.1)	70 (37.6)	
2, n (%)	83 (35.8)	15 (32.6)	68 (36.6)	
3, n (%)	61 (26.3)	13 (28.3)	48 (25.8)	
Left main trunk stenosis > 50%, n (%)	19 (8.2)	2 (4.3)	17 (9.1)	0.380
Chronic total occlusion in other vessels, n (%)	16 (6.9)	4 (8.7)	12 (6.5)	0.530
Intra-aortic balloon pump, n (%)	9 (3.9)	2 (4.3)	7 (3.8)	0.854
Veno-arterial extracorporeal membrane oxygenation, n (%)	7 (3.0)	1 (2.2)	6 (3.2)	1.000
Atropine, n (%)	68 (29.3)	15 (32.6)	53 (28.5)	0.583
Norepinephrine before revascularization, n (%)	60 (25.9)	17 (37.0)	43 (23.1)	0.055
Dopamine before revascularization, n (%)	6 (2.6)	3 (6.5)	3 (1.6)	0.094
Dobutamine before revascularization, n (%)	4 (1.7)	4 (8.7)	0 (0.0)	0.001
Culprit lesion				0.341
1, n (%)	60 (25.9)	16 (34.8)	44 (23.7)	
2, n (%)	84 (36.2)	17 (37.0)	67 (36.0)	
3, n (%)	63 (27.2)	11 (23.9)	52 (28.0)	
4AV, n (%)	19 (8.2)	2 (4.3)	17 (9.1)	
4PD, n (%)	6 (2.6)	0 (0.0)	6 (3.2)	
Initial TIMI flow grade				0.024
0, n (%)	156 (67.2)	39 (84.8)	117 (62.9)	
1, n (%)	18 (7.8)	3 (6.5)	15 (8.1)	
2, n (%)	27 (11.6)	3 (6.5)	24 (12.9)	
3, n (%)	31 (13.4)	1 (2.2)	30 (16.1)	
Final TIMI flow grade				0.331
0, n (%)	2 (0.9)	0 (0.0)	2 (1.1)	
1, n (%)	3 (1.3)	1 (2.2)	2 (1.1)	
2, n (%)	10 (4.3)	0 (0.0)	10 (5.4)	
3, n (%)	217 (93.5)	45 (97.8)	172 (92.5)	
TIMI Thrombus grade				0.001
1, n (%)	36 (15.5)	0 (0.0)	36 (19.4)	
2, n (%)	16 (6.9)	1 (2.2)	15 (8.1)	
3, n (%)	16 (6.9)	2 (4.3)	14 (7.5)	
4, n (%)	8 (3.4)	4 (8.7)	4 (2.2)	
5, n (%)	156 (67.2)	39 (84.8)	117 (62.9)	
Approach site				< 0.001
Radial, n (%)	148 (63.8)	18 (39.1)	130 (69.9)	
Brachial, n (%)	3 (1.3)	0 (0.0)	3 (1.6)	
Femoral, n (%)	81 (34.9)	28 (60.9)	53 (28.5)	
Size of guide catheter				0.043
6 Fr, n (%)	174 (75.0)	30 (65.2)	144 (77.4)	
7 Fr, n (%)	57 (24.6)	15 (32.6)	42 (22.6)	
8 Fr, n (%)	1 (0.4)	1 (2.2)	0 (0.0)	
Thrombectomy, n (%)	76 (32.8)	27 (58.7)	49 (26.3)	< 0.001
Final PCI procedures				0.291
POBA, n (%)	13 (5.6)	0 (0.0)	13 (7.0)	
Thrombectomy, n (%)	6 (2.6)	1 (2.2)	5 (2.7)	
Thrombectomy and POBA, n (%)	4 (1.7)	0 (0.0)	4 (2.1)	
Drug-coated balloon, n (%)	2 (0.9)	0 (0.0)	2 (1.1)	
Drug-eluting stent implantation, n (%)	194 (83.6)	41 (89.1)	153 (82.3)	
Bare-metal stent implantation, n (%)	11 (4.7)	4 (8.7)	7 (3.8)	
Bougie only, n (%)	2 (0.9)	0 (0.0)	2 (1.1)	
Right coronary artery				0.351
Dominance, n (%)	225 (97.0)	46 (100.0)	179 (96.2)	
Balanced dominance, n (%)	7 (3.0)	0 (0.0)	7 (3.8)	
Number of #4AV				0.167
0, n (%)	8 (3.4)	0 (0.0)	8 (4.3)	
1, n (%)	72 (31.0)	17 (37.0)	55 (29.6)	
2, n (%)	69 (29.7)	10 (21.7)	59 (31.7)	
3, n (%)	63 (27.2)	17 (37.0)	46 (24.7)	
4, n (%)	16 (6.9)	1 (2.2)	15 (8.1)	
5, n (%)	4 (1.7)	1 (2.2)	3 (1.6)	
QCA lesion length, mm	15.5 ± 9.1 (230/232)	16.6 ± 9.4	15.3 ± 9.0 (184/186)	0.380
QCA reference diameter, mm	2.9 ± 0.7 (230/232)	2.9 ± 0.6	2.9 ± 0.7 (184/186)	0.814
Stent length, mm	26.6 ± 12.6 (207/207)	28.4 ± 12.3 (45/45)	26.1 ± 12.7 (162/162)	0.248
Stent diameter, mm	2.9 ± 0.4 (207/207)	3.0 ± 0.3 (45/45)	2.9 ± 0.4 (162/162)	0.126
Door to balloon time, min	76.2 ± 36.7 (217/233)	79.7 ± 42.0 (44/46)	75.2 ± 35.3 (173/186)	0.798
Fluoroscopy time, min	25.3 ± 16.9 (225/232)	28.9 ± 20.5 (44/46)	24.5 ± 15.9 (181/186)	0.033
Amount of contrast agent, mL	113.1 ± 37.8 (227/232)	112.1 ± 36.9 (45/46)	113.3 ± 38.1 (182/186)	0.582
Revascularization strategy to multi-vessel disease				0.594
Single vessel disease, n (%)	88 (37.9)	18 (39.1)	70 (37.6)	
Complete revascularization for multi-vessel disease during the index hospitalization, n (%)	70 (30.2)	13 (28.3)	57 (30.6)	
Complete revascularization for multi-vessel disease after discharge of the index hospitalization, n (%)	36 (15.5)	5 (10.9)	31 (16.7)	
Incomplete revascularization for multi-vessel disease, n (%)	38 (16.4)	10 (21.7)	28 (15.1)	

Data were expressed as mean ± SD or numbers (percentages). A Student’s *t* test was used for normally distributed continuous variables, and Mann–Whitney *U* test was used for abnormally distributed continuous variables. A Chi-square test was used for categorical variables.

Abbreviations: *BMS* bare metal stent, *CABG* coronary artery bypass grafting, DCB drug-coated balloon, DES drug eluting stent, *PCI* percutaneous coronary intervention, *POBA* Percutaneous old balloon angioplasty, *TIMI* thrombolysis in myocardial infarction.

**Fig 2:**
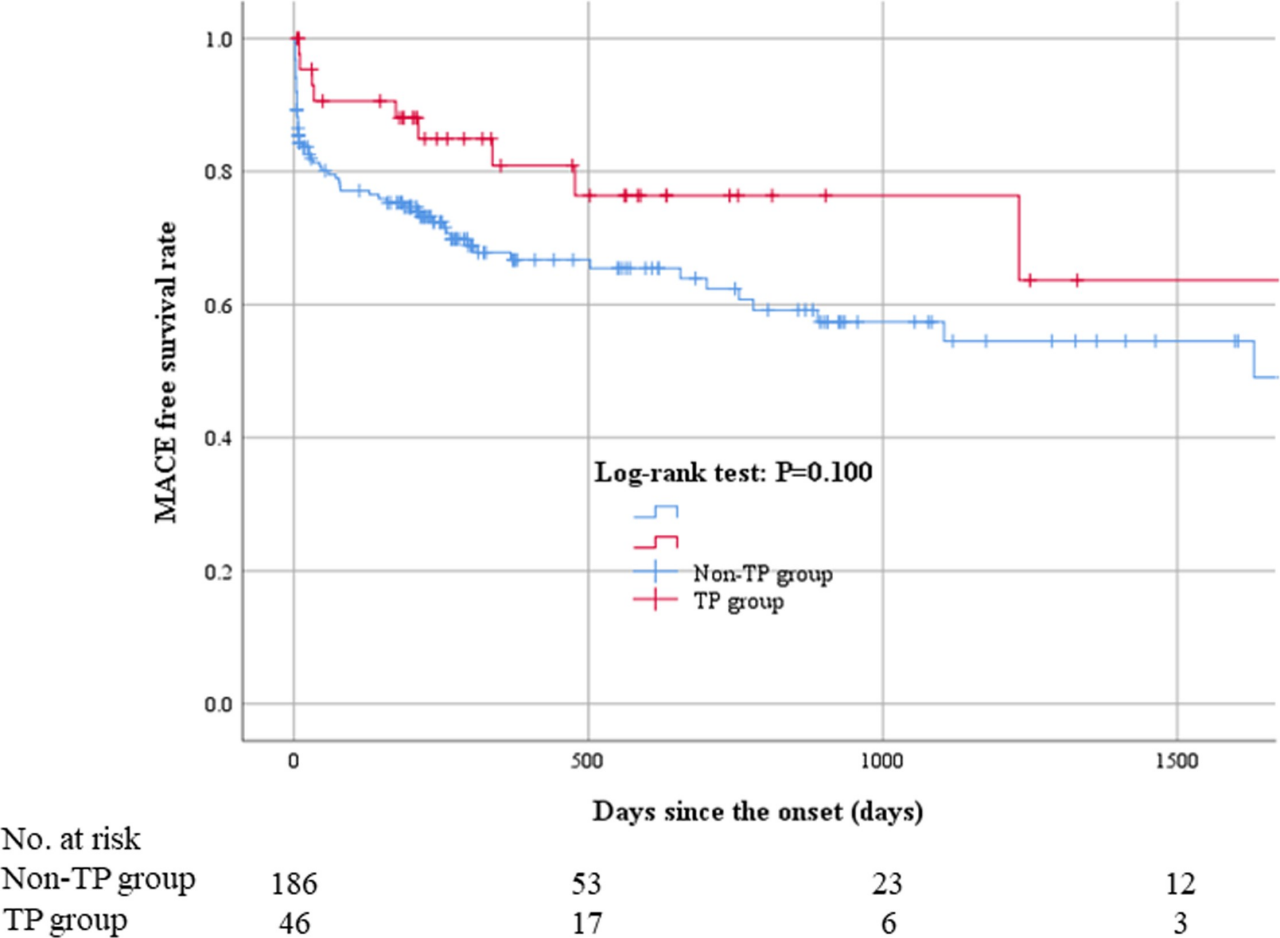
Kaplan–Meier curves for MACE. MACE were not different between the 2 groups (P = 0.100). Abbreviations: MACE = major adverse cardiovascular events.

The comparisons of clinical outcomes between the TP and non-TP groups are shown in Table 4. The frequency of right ventricular infarction was significantly higher in the TP group (19.6%) than in the non-TP group (7.5%) (p = 0.024). The incidence of in-hospital death was similar between the 2 groups (p = 1.000). The length of hospital and CCU stay were longer in the TP group (11.0 ± 7.8 and 3.7 ± 2.5) than in the non-TP group (9.6 ± 9.1 and 3.4 ± 3.6) (p = 0.014 and 0.015, respectively). Median follow-up duration was 316.5 days (Q1: 196.25 days—Q3: 876.25 days). The Kaplan-Meier curves for MACE are shown in Fig 2. MACE were not different between the 2 groups (P = 0.100).

**Table 4:** The comparison of clinical outcomes between the temporary pacing group and the non-temporary pacing group.

	**All (n = 232)**	**TP group (n = 46)**	**Non-TP group (n = 186)**	***P* value**
In-hospital outcomes				
Right ventricular infarction	23 (9.9)	9 (19.6)	14 (7.5)	0.024
Peak CK, mg/dL	1963.3 ± 1995.7	2384.4 ± 2633.3	1859.1 ± 1797.6	0.302
Peak CK-MB, mg/dL	180.1 ± 186.7	185.7 ± 183.5	178.7 ± 188.0	0.782
Left ventricular ejection fraction, %	53.1 ± 10.7 (173/232)	54.0 ± 8.8 (30/46)	52.9 ± 11.0 (143/186)	0.805
In-hospital death, n (%)	11 (4.7)	2 (4.3)	9 (4.8)	1.000
Length of hospital stay, days	9.9 ± 8.9	11.0 ± 7.8	9.6 ± 9.1	0.014
Length of CCU stay, days	3.5 ± 3.4	3.7 ± 2.5	3.4 ± 3.6	0.015
Permanent pacemaker implantation during the admission, n (%)	1 (0.4)	0 (0.0)	1 (0.5)	1.000
Long-term clinical outcomes				
Follow-up duration	543.6 ± 512.8	505.1 ± 475.9	553.1 ± 522.2	0.654
MACE, n (%)	73 (31.5)	10 (21.7)	63 (33.9)	0.113
All cause death, n (%)	21 (9.1)	4 (8.7)	17 (9.1)	1.000
Re-admission for heart failure, n (%)	11 (4.7)	4 (8.7)	7 (3.8)	0.235
No fatal MI, n (%)	13 (5.6)	2 (4.3)	11 (5.9)	1.000
TVR, n (%)	43 (18.5)	2 (4.3)	41 (22.0)	0.006

MACE indicates major adverse cardiovascular events: composite of all cause death, no fatal MI, TVR and re-admission for heart failure.

Data were expressed as mean ± SD or numbers (percentages). A Student’s *t* test was used for normally distributed continuous variables, and Mann–Whitney *U* test was used for abnormally distributed continuous variables. A Chi-square test was used for categorical variables.

Abbreviations: *CCU* coronary care unit, *CK* creatine kinase, *CK-MB* creatine kinase MB, *MACE* major adverse cardiovascular events, *MI* myocardial infarction, *TVR* target vessel revascularization.

We performed univariate and multivariate logistic regression analysis to find factors associated with TP (Table 5). Statin use at admission (OR 0.230, 95% CI 0.062–0.860, p = 0.029), HAVB at admission (OR 9.950, 95% CI 4.099–24.152, p<0.001), and TIMI-thrombus grade ≥3 (OR 10.762, 95% CI 1.385–83.635, p = 0.023) were significantly associated with TP.

**Table 5:** Determinants of temporary pacing: Univariate and multivariate logistic regression analysis.

Dependent variable: temporary pacing						
	Univariate logistic regression analysis			Multivariate logistic regression analysis		
	OR	95% CI	P value	OR	95% CI	P value
Independent variables						
Age (10 year increase)	0.986	0.784–1.240	0.903			
Male (vs. female)	1.183	0.545–2.569	0.671			
Hypertension	0.900	0.445–1.823	0.771			
Diabetes mellitus	0.741	0.378–1.454	0.383			
Dyslipidemia	0.476	0.244–0.931	0.030			
Aspirin	0.356	0.104–1.222	0.101			
Thienopyridine	0.216	0.028–1.665	0.141			
Statin at admission	0.199	0.059–0.671	0.009	0.230	0.062–0.860	0.029
History of previous myocardial infarction	0.322	0.073–1.419	0.134			
High-degree atrioventricular block at admission	15.909	6.870–36.843	< 0.001	9.950	4.099–24.152	< 0.001
Shock at admission	3.266	1.541–6.920	0.002	2.099	0.833–5.293	0.116
Systolic blood pressure at admission (10 mmHg)	0.835	0.745–0.937	0.002			
Diastolic blood pressure at admission (10 mmHg)	0.729	0.607–0.875	0.001			
Heart rate at admission (5 bpm)	0.680	0.598–0.774	< 0.001			
Atropine, n (%)	1.214	0.607–2.430	0.583			
QCA lesion length (5 mm increase)	1.064	0.902–1.256	0.459			
QCA reference diameter (0.5 mm)	1.003	0.804–1.250	0.982			
Right coronary artery dominant*	-	-	-			
TIMI-thrombus grade ≥3	17.000	2.283–126.577	0.006	10.762	1.385–83.635	0.023
Thrombectomy	3.973	2.030–7.776	<0.001			

Univariate logistic regression analysis was performed to identify variables that had marginal association with temporary pacing. All variables that had marginal association in univariate analysis were adopted as independent variables in multivariate logistic regression analysis.

*Univariate and multivariate logistic regression analysis cannot be conducted in “Right coronary artery dominant” because all of the patient in the TP group have dominant right coronary artery.

Abbreviations: *eGFR* estimated glomerular filtration rate, *PCI* percutaneous coronary intervention.

## Discussion

The present study included 232 inferior STEMI patients, and divided those into 46 patients (19.8%) who required TP and 186 patients (80.2%) who did not. The TP group showed a higher incidence of RV infarction, and a longer period of hospital stay compared to the non-TP group, but the incidence of in-hospital death and long-term MACE was not different between the 2 groups. We found that statin use at admission, HAVB at admission, and TIMI-thrombus grade ≥3 were significantly associated with TP. It may be important for interventional cardiologists to recognize those factors to prepare TP in emergent situations.

The earlier studies in the thrombolytic era reported that HAVB in inferior AMI was associated with older age, larger infarct size, female predominance, and higher mortality [25–27]. Since primary PCI has replaced thrombolysis in the treatment of STEMI in most developed countries, the incidence of HAVB has been decreasing and the mortality rate has been significantly improved. However, the presence of HAVB was still a significant prognostic factor for a lower chance of survival [3, 4, 28]. Indeed, the TP group showed a higher rate of RV infarction and a longer period of hospital compared to the non-TP group in the present study, but those reported factors including age, infarct size, sex, and mortality were not different between the 2 groups in the present study.

We should discuss why TIMI-thrombus grade was closely associated with TP in inferior STEMI. Tanboga et al. [29] reported high thrombus burden in patients with STEMI was associated with distal embolization and impaired post-procedural epicardial and myocardial perfusion. Thus, in patients with high thrombus burden, the incidence of distal embolization to the territory of cardiac conduction system might be high, which lead to bradycardia requiring TP. However, most patients with TP underwent the insertion of TP before revascularization in the present study, which suggests that the distal embolization caused by PCI might not be associated with insertion of TP. High thrombus burden itself might be the cause of bradycardia requiring TP. Another possibility was that high thrombus burden was not the cause of bradycardia, but the effect of bradycardia. Bradycardia might provoke the stagnation of coronary flow, which results in thrombus formation. Our retrospective study could not provide an answer whether high thrombus burden was either a cause or an effect of bradycardia.

In our study, statin use at admission was inversely associated with insertion of TP in patients with inferior STEMI. Early statin administration in patients with AMI is known to reduce the prevalence of positive vascular remodeling and to alter plaque components such as the amount of necrotic core and fibro-fatty plaque. Furthermore, chronic statin treatment is reported to reduce positive remodeling in the culprit lesions of patients with ACS [30–33]. In this way, statin before admission might stabilize the plaque of the culprit lesions, and could consequently reduce the thrombus burden.

Clinical implications of the present study should be noted. In general, if a patient with inferior STEMI comes to an emergency room with shock caused by HAVB, we would not hesitate to insert TP. However, since insertion of TP is a time-consuming and invasive procedure, the decision to insert TP is sometimes difficult for interventional cardiologists. When we cannot make a quick decision whether to insert TP for patients with inferior STEMI, information regarding statin treatment before admission or TIMI-thrombus grade from initial coronary angiography may be helpful. Specific techniques such as distal protection devices may be considered to prevent distal embolization and subsequent bradycardia for patients with high thrombus burden [34]. Moreover, when a patient with inferior STEMI requires TP, we should be careful about the occurrence of RV infarction as a possible complication.

## Study limitations

Our study has several limitations. First, since our study was designed as a single-center, retrospective observational study, there is a risk of selection bias. Second, since our study was conducted with a relatively small number of patients, especially only 46 patients in the TP group, there is a possibility of beta errors. Third, the decision whether or not to use TP finally depended on the interventional cardiologist’s discretion. Some interventional cardiologists might not insert TP for patients with HAVB, whereas other interventional cardiologists might insert TP in a preventive manner for patients without bradycardia. In order to minimize this limitation, we excluded the patients with TP whose TP was not activated. Although TP is class I recommendation in clinical guidelines for symptomatic bradyarrhythmias unresponsive to medical treatment in patients with STEMI [35], the literatures supporting TP for inferior STEMI are sparse. Since it may be ethically difficult to randomly allocate patients with symptomatic bradyarrhythmias into the non-TP group, further well-conducted retrospective studies including registry data are warranted to confirm our results.

## Conclusions

Statin use at admission, HAVB at admission, and TIMI-thrombus grade ≥3 were closely associated with insertion of TP in patients with inferior STEMI. Patients with TP showed a higher incidence of RV infarction and a longer period of hospital stay than patients without, but the incidence of in-hospital death and long-term MACE was not different.

## Supporting information

S1 Table The detail regarding the catecholamine and mechanical circulatory support use by each bradyarrhythmia. (DOCX)

S1 Dataset (XLSX)
