# Supplementary material for: Factors associated with temporary pacing insertion in patients with inferior ST-segment elevation myocardial infarction
Source: PLoS One. 2021 May 3;16(5):e0251124. doi: 10.1371/journal.pone.0251124 (PMC8092657; doi:10.1371/journal.pone.0251124)
Supplement: S1 Table — (DOCX) [file pone.0251124.s001.docx]

**S1 Table. The detail regarding the catecholamine and mechanical circulatory support use by each bradyarrhythmia.**

|  | All (n = 46) | HAVB (n = 32) | SSS (n = 11) | Atrial fibrillation with bradycardia (n = 2) | bradycardia (details unknown) (n = 1) |
| --- | --- | --- | --- | --- | --- |
| Intra-aortic balloon pump, n (%) | 2 | 2 | 0 | 0 | 0 |
| Veno-arterial extracorporeal membrane oxygenation, n (%) | 1 | 1 | 0 | 0 | 0 |
| Atropine, n (%) | 15 | 10 | 5 | 0 | 0 |
| Norepinephrine before revascularization, n (%) | 17 | 12 | 5 | 0 | 0 |
| Dopamine before revascularization, n (%) | 3 | 3 | 0 | 0 | 0 |
| Dobutamine before revascularization, n (%) | 4 | 2 | 2 | 0 | 0 |
